# Supplementary material for: SAGA Complex Subunit Hfi1 Is Important in the Stress Response and Pathogenesis of Cryptococcus neoformans
Source: J Fungi (Basel). 2023 Dec 15;9(12):1198. doi: 10.3390/jof9121198 (PMC10744473; doi:10.3390/jof9121198)
Supplement: Supplementary file 1 [file jof-09-01198-s001.zip › jof-2712598-SI.pdf]

Supplementary materials

Table S1: Strains used in this study.

Strain	Function	*Species*	Gene	Background	Selective marker	Source
H99O	Wild-type	*C. neoformans*		Clinical		(Janbon *et al.*, 2014)
CJS24	*hfi1*Δ	*C. neoformans*	*HFI1*	H99O	*NEO*	This study
KY06	*HFI1* complementation	*C. neoformans*	*HFI1*	CJS24	*NAT*	This study
Mach1	Cloning	*E. coli*		Mach1	*bla*	Thermo Scientific

Table S2: Primers used in this study.

Fragment / Gene	ID	Sequence
HFI1 – 5' flanking region	UQ5699	5′- CTATAGGGCGAATTGGGTACCCTTTGCTAACTGCCGTGCTGA -3′
	UQ5700	5′- TCCAGCTCACATCCTCGCAGCTAGAAGTGTGGAAAAAGGCCG -3′
HFI1 – 3' flanking region	UQ5702	5′- CCGTGTTAATACAGATAAACCGGAGGTGTTTGGTTGGTGTAG -3′
	UQ5704	5′- CCGCTCTAGAACTAGTGGATCACCTCTGGAGATGGAGATAGG -3′
HFI1 H99O gene overhang with pSDMA25	UQ5999	5′- GGGTACCGGGCCCCCCCTCGACTTTGCTAACTGCCGTGCTGA -3′
	UQ6000	5′- GTGGCGGCCGCTCTAGAACTAGACCTCTGGAGATGGAGATAGG -3′
HFI1 – NEO deletion plasmid sequencing	UQ6001	5′- GAGCATCGATGTCCTTCAAAC-3′
	UQ6002	5′- CTTCCAACCTACATCAAATAG-3′
	UQ6003	5′- CAGCAGCAGCGGAAAAGGATAT -3′
	UQ6004	5′- TCCACAGGCTAATGACAGCAC -3′
	UQ6005	5′- GTACGGTTATAATCTCACAGC -3′
HFI1 in pSDMA25 sequencing	UQ6001	5′- GAGCATCGATGTCCTTCAAAC -3′
	UQ6003	5′- CAGCAGCAGCGGAAAAGGATAT -3′
	UQ5746	5′- AAATATTAACGCTTACAATTT -3′
	UQ5747	5′- CGACTGGAAAGCGGGCAGTGA -3′
	UQ6002	5′- CTTCCAACCTACATCAAATAG -3′
	UQ6004	5′ TCCACAGGCTAATGACAGCAC -3′
	UQ6005	5′- GTACGGTTATAATCTCACAGC -3′
HFI1 RT-PCR	UQ6258	5′- GTTAAAAGGCGATCATTTACACC -3′
	UQ6259	5′- CGTTGTTGGAGGGAGGA -3′
ADA2 RT-PCR	UQ6260	5′- CAGAGTATCGGGCTATACAAGC -3′
	UQ6261	5′- CTTCGGCAGTTTGCAACTTT -3′
NGG1 RT-PCR	UQ6300	5′- AGTGGATGTGGTTGATTTGG -3′
	UQ6301	5′- TTATTCGGGTCAAACTCTTCG -3′
CHD1 RT-PCR	UQ6262	5′- CTTGCCGATGAAATGGGTCTC -3′
	UQ6263	5′- GAGGGACAACAACAAGGAAAGG -3′
GCN5 RT-PCR	UQ6264	5′- CCGAACAAATCAAGGGTTACG -3′
	UQ6265	5′- TGTCCGCATAAGTCAGAAAGA -3′
SGF11 RT-PCR	UQ6202	5′- ATCAACTGGTGTGGGTAGTG -3′
	UQ6203	5′- ATATCGGTTGGAGGCAATAGG -3′
SGF29 RT-PCR	UQ6266	5′- CAAGTGGCGATGATTGGATTC -3′
	UQ6267	5′- GGGTAGTGTTGTATGTGTTGC -3′
SGF73 RT-PCR	UQ6268	5′- CCGGAAATGAAGAGGGAAGG -3′
	UQ6269	5′- CATGATTACACACCCGTCGAT -3′
SPT3 RT-PCR	UQ6270	5′- GGCACAGGCCCAAGTAAA -3′
	UQ6271	5′- CTCCAAACACAAACATCATCTGC -3′
SPT7 RT-PCR	UQ6302	5′- GTAGATCATCTGGATGGGTTGG -3′
	UQ6303	5′- GGCATGTAGAATGATCTCCTCG -3′
SPT8 RT-PCR	UQ6272	5′- GATGGAACCGTCAGAGAATGG -3′
	UQ6273	5′- GGAAGAGAGTTGGGCTTTGT -3′
SPT20 RT-PCR	UQ6274	5′- GATGGTCCAATGAAGCCATTCC -3′
	UQ6275	5′- GATGGGTGGTTGGATGTCATAAAG -3′
SUS1 RT-PCR	UQ6276	5′- GTTGATGTTACGGTCGATGAG -3′
	UQ6277	5′- AGTAGCTTTTGTATTCGCTCC -3′

TAF5 RT-PCR	UQ6278	5′- CGATGGTGGATGTCAGTGAA -3′
	UQ6279	5′- AGATTATCCAGTTCAGCCTTCC -3′
TAF6 RT-PCR	UQ6280	5′- CGAGTATTGGGCATTTGGTAGA -3′
	UQ6281	5′- GGAGGAGTTGATGCATGTATGG -3′
TAF9 RT-PCR	UQ6222	5′- GAAGCTCCTCCAAGAGATTACC -3′
	UQ6223	5′- GTAGACGGACGAGGTCAAATG -3′
TAF10 RT-PCR	UQ6282	5′- GGTACAATCGTCAATGTGTCC -3′
	UQ6283	5′- ATTAGGTGTGTAGGGTTCATCT -3′
TAF12 RT-PCR	UQ6304	5′- GGCTTTCCGCCAAAGTACC -3′
	UQ6305	5′- GGTTCTGATGTTGGGCGTTATT -3′
TRA1 RT-PCR	UQ6284	5′- TTCTTTCTGTCGGGATTCTCG -3′
	UQ6285	5′- CGCCTTGACGTGTGTAAGATA -3′
UBP8 RT-PCR	UQ6286	5′- GACAAGTTGTCGTGGTCTGC -3′
	UQ6287	5′- TGCTTTGAGAAGCGGATTATGG -3′
ACT1 RT-PCR	UQ482	5′- CCTACAACTCTATCATGAAGTGTGATCTC -3′
	UQ728	5′- TCTGCATACGGTCGGCAATAC -3′
TUB2 RT-PCR	UQ484	5′- AGTCGCTTTTCAAGCGTATCG -3′
	UQ729	5′- GGATTCGGCTTCAGAGAATTCA -3′
GPD1 RT-PCR	UQ486	5′- GTCTCTACTGATTTCGTTGGCACTAC -3′
	UQ730	5′- GTAACCGTACTCATTGTCATACCAGCTA -3'
HHT1 RT-PCR	UQ488	5′- GAAATCCGACGATACCAGAAGTCTAC -3′F
	UQ731	5′- GGAATCGAAGGTCGGTCTTG -3′
*sgf29*Δ testing	UQ3574	5′- CTGTAGAGCTCCTCGAAATAC -3′
	UQ3575	5′- CCATACCTAGGCCATCCATAC -3′

Table S3: Plasmids used in this study.

Strain	Function	Plasmid backbone	Marker	Selective marker	Source
pJAF1	*NEO* marker	pBluescript SK(-)	*NEO*	*bla*	Fraser *et al.* (2003)
pSDMA25	Safe Haven 1	pBluescript SK(-)	*NAT*	*bla*	Arras *et al.* (2015)
**pCJS48**	*hfi1*Δ	pBluescript SK(-)	*NEO*	*bla*	This study
pKY04	*HFI1* complementation	pSDMA25	*NAT*	*bla*	This study

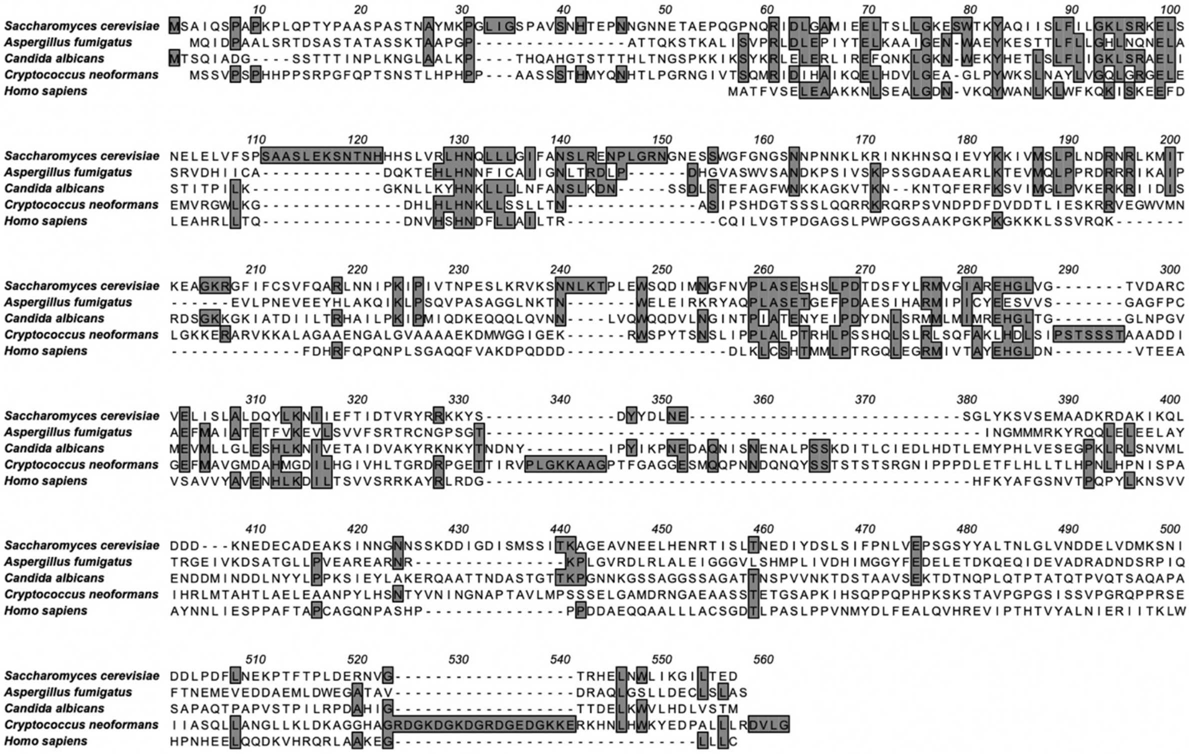

Figure S1: Protein alignments of Hfi1 from *Saccharomyces cerevisiae*, *Aspergillus fumigatus*, *Candida albicans*, *Cryptococcus neoformans* and *Homo sapiens*. Numbering for is based on the *C. neoformans* sequence.

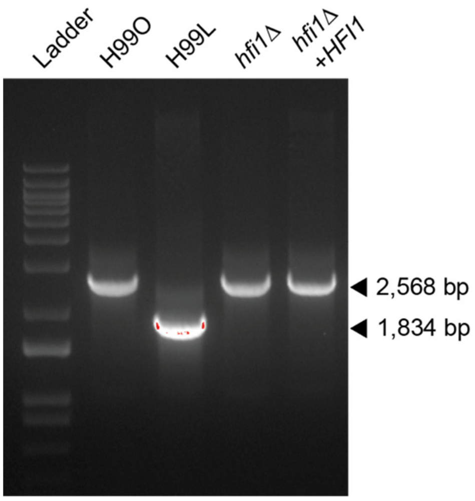

Figure S2: H99L *sgf29*Δ allele background verification. PCR using primers UQ3574 and UQ3575 amplifies a 2568 bp *SGF29* fragment from wild-type (H99O, lane 2) and a 1834 bp band from strains bearing the H99L *sgf29*Δ allele (H99L, lane 3) easily visible on a 1% agarose TAE gel. Lane 1, Invitrogen 1 Kb Plus DNA Ladder. Lane 3 and Lane 4, a 2568 bp fragment from the mutant and complemented strains.

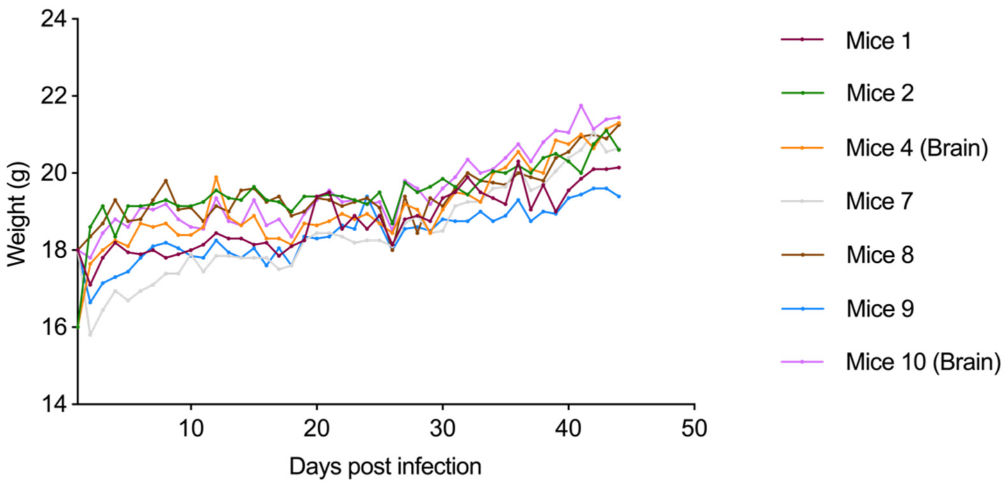

Figure S3: Changing of weight for seven mice during the experiment. Mice 1, 2, 7, 8, 9 had colonies identified in the lung. Mice 4 and 10 had colonies identified in both lung and brain.
